# Carotenoid biosynthesis in *Prunus *species: from pathway and accumulation structure to diverse pigmentation

**DOI:** 10.1186/s43897-025-00188-6

**Published:** 2026-01-04

**Authors:** Naila Mir Baz, Jiahui Wang, Xulei Zhao, Asia Maqbool, Caizhen Gao, Pengfei Wang, Haijiang Chen, Hongbo Cao

**Affiliations:** College of Horticulture, Hebei Agricultural University, Baoding, 071000 China

**Keywords:** Carotenoids, *Prunus*, Biosynthesis, Genetic variability, Chromoplast

## Abstract

*Prunus* species, also known as stone fruits, include several eye-catching fruits such as cherries, plums, apricots, peaches, nectarines, etc., which have significant economic value and are widely cultivated worldwide. Carotenoids are important secondary metabolites contributing to stone fruits' aesthetic appeal and nutritional value. Carotenoids contribute hues ranging from pastel yellow to rich orange in *Prunus* fruits. Carotenoids accumulate in *Prunus* tissues through the action of chromoplasts, particular structures that store and stabilize these pigments, giving rise to their vibrant colors. The diversity in carotenoid types and levels among *Prunus* species and cultivars leads to diverse tissue colors, reflecting their genetic diversity and evolutionary adaptations. The most important genes related to coloration are *PSY, LCYB/E*, and *BCH1*, which are responsible for carotenoid biosynthesis, whereas *CCDs* and *NCEDs* are involved in the degradation of carotenoids. *PSY* leads to increased carotenoid accumulation, providing yellow and orange pigmentation. *LCYB* involved in β-carotene accumulation results in an orange color. *LCYE* can lead to lutein biosynthesis and contribute to yellow coloration. *BCH1* contributes to yellow pigmentation. *CCD4* plays an essential role in the flesh color of the fruit, leading to white flesh in *Prunus* fruits, especially peaches. *NCED* is involved in abscisic acid formation by degrading carotenoids. Despite the importance of carotenoids, the connection between carotenoid profiles and the diversity of *Prunus* fruits has received little attention in the past. This review outlines the present knowledge regarding the molecular diversity mechanisms of the carotenoid biosynthesis pathway in *Prunus* fruits.

## Introduction

Carotenoids are key plant metabolites that contribute significantly to the aroma and pigmentation of *Prunus* fruits. As secondary plant metabolites, they are significant for their wide distribution, structural variety, and numerous functions. Belonging to the category of isoprenoids, these metabolites are produced by all photosynthetic organisms and some non-photosynthetic prokaryotes, including fungi (Zia-Ul-Haq 2021). They can absorb light energy when it is in an energized state (Llorente et al. 2017). Carotenoids have multiple functions in plants as they help in the process of photosynthesis (Hashimoto et al. 2016; Zhang et al. 2018) and are accessory pigments that help pollination by attracting insects and are precursors of abscisic acid (Hou et al. 2016). Carotenoids have intense biological activity and regulate fruit flavor and color. Carotenoids come in various colors, i.e., colorless to yellow, orange, and red. Color changes and sugar metabolism are crucial physiological processes of fruits that directly influence the scent and flavor of fruits (Lin et al. 2023). Biochemical techniques in the 1960 s and molecular approaches in the 1980 s were employed to explain the carotenoid biosynthesis pathway in the mid-twentieth century. Specific advancements were made in the 1990 s to identify genes and enzyme pathways.

**Fig. 1:**
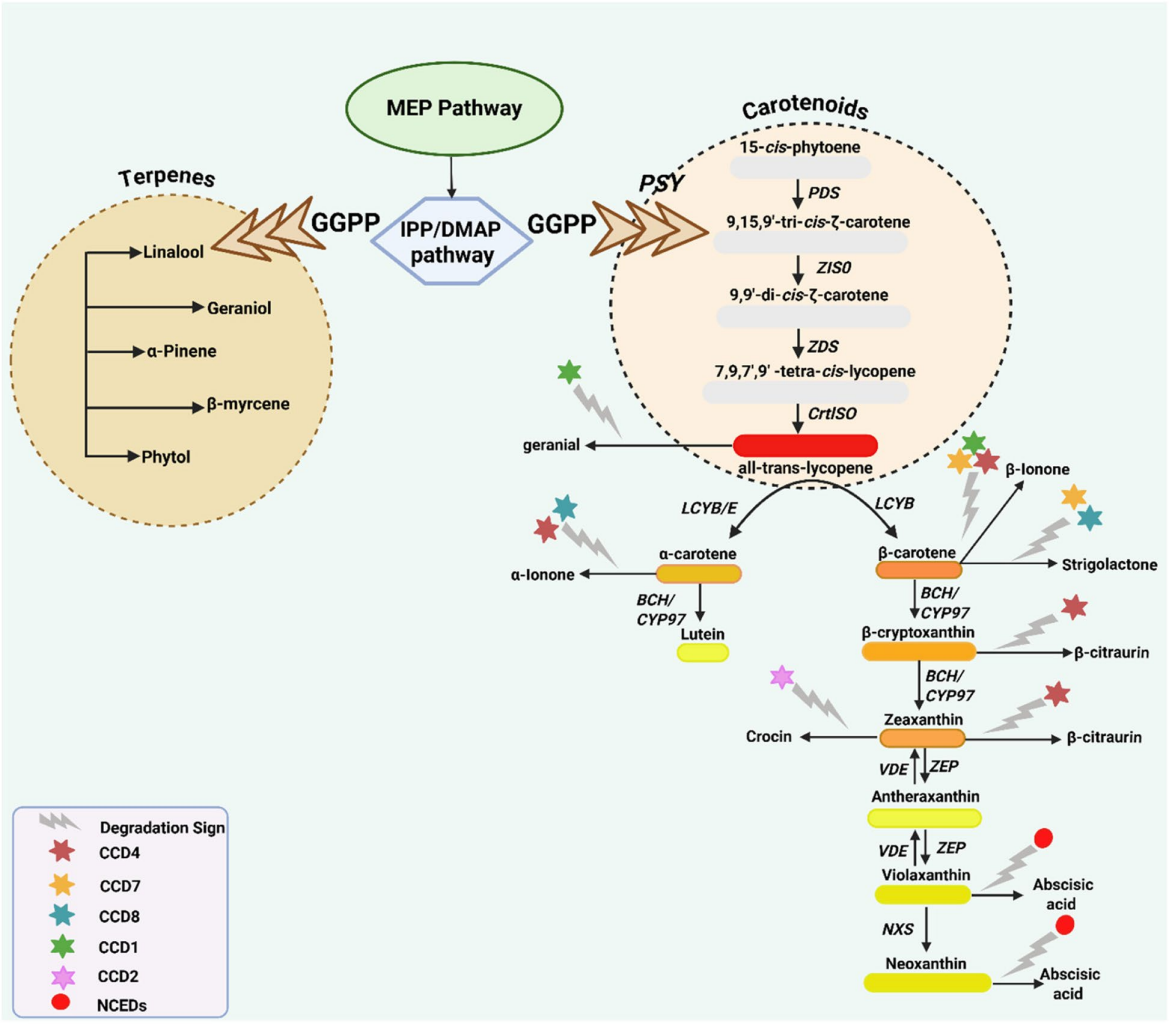
The carotenoid biosynthetic pathway in *Prunus* fruits. The enzymes involved in each reaction are represented by arrows that show the enzymatic alterations. GGPP: Geranyl–Geranyl Pyrophosphate Synthase; PSY: Phytoene Synthase; PDS: Phytoene Desaturase; ZISO: ζ-carotene Isomerase; ZDS: ζ-carotene Desaturase; CrtISO: Carotenoid Isomerase; LCYB: Lycopene α-cyclase; LCYE: Lycopene ε-cyclase; BCH: β-carotene Hydroxylases; CYP97: Cytochrome P450, Family 97; ZEP: Zeaxanthin Epoxidase; VDE: Violaxanthin de-epoxidase; NXS: Neoxanthin synthase; NCED: 9-cis-epoxy carotenoid dioxygenase. Stars represent the carotenoid cleavage dioxygenases (CCDs). Different colors represent various *CCD*s

Furthermore, despite the research's encouragement of regulatory difficulties, most biosynthetic genes have recently been identified (Cazzonelli and Pogson 2010). The biosynthesis process of the carotenoid has been thoroughly investigated in a variety of plants, including the model plant *Arabidopsis* (Lu and Li 2008; Shumskaya and Wurtzel 2013; Baranski and Cazzonelli 2016; Sun et al. 2022) and a series of processes, including desaturation, cyclization, hydroxylation, and epoxidation accomplish it. The C5 IPP in plastids is the starting point for the carotenoid biosynthesis pathway, where the final product accumulates (Wang et al. 2023a). Briefly, the carotenoid biosynthesis pathway (Fig. 1) starts with the Geranylgeranyl pyrophosphate (GGPP) and undergoes a series of enzymatic reactions to be converted into lycopene. The condensation of three IPPs and one DMAPP results in the formation of C20 GGPP. The initial C40 carotenoid, phytoene, is produced through the head-to-head coupling of two GGPP molecules catalyzed by phytoene synthase (García-Gómez et al. 2020a, b; Yan et al. 2020). Following the formation of phytoene, phytoene desaturase (PDS) and ζ-carotene desaturase (ZDS) catalyze the addition of conjugated double bonds to the intermediate pigments. This process results in the production of phyto-fluene (a colorless pigment), ζ-carotene (a pale yellow pigment), neurosporene (an orange-yellow pigment), and red lycopene (Nisar et al. 2015; Zheng et al. 2023a, b).

Furthermore, LCYB and LCYE catalyze the cyclization of lycopene, synthesizing two distinct kinds of carotenes with one or two rings of the β-type or ε-type. Lycopene is transformed into β-carotene and β-cryptoxanthin when only LCYB is active in this stage; it is then further metabolized to zeaxanthin via two hydroxylation steps by carotene β-hydroxylase (BCH), producing a range of oxygenated derivatives known as xanthophylls (García-Gómez et al. 2020a, b; Wang et al. 2023a). Zeaxanthin epoxidase (ZEP) catalyzes the epoxidation of the β-ring of zeaxanthin at positions C5, 6, and C5′, 6′ to produce antheraxanthin and violaxanthin, which are then metabolized to neoxanthin-by-neoxanthin synthase (NXS) (Xi et al. 2020; Zhou et al. 2021). NCED breaks down 9-cis-violaxanthin and 9-cis-neoxanthin into xanthoxin (C15), which is then transformed into ABA via an intermediate ABA aldehyde. Apo-carotenoid molecules are produced due to various levels of carotenoid breakdown by the enzyme carotenoid cleavage dioxygenase (CCD) (Borovsky et al. 2004; Xi et al. 2020; Liang et al. 2021).

The carotenoid biosynthesis pathway is well established in *Prunus* species and other common plants such as *Arabidopsis* and tomato (Deng et al. 2023). Carotenoid assembly continues to take place within plastids. Diverse plastid types have radically diverse capacities and abilities for accumulating carotene. A histological examination revealed that the fruit's carotenoids came from two distinct sources. They originated from chloroplasts that transformed into chromoplasts in the fruit peel, while carotenoids in the fruit flesh possibly came from pro-plastids (Lu et al. 2019). Carotenoid synthesis in the chloroplast is often a constitutive process that produces a sealed carotenoid outline that is regulated by neoxanthin (10–15%), violaxanthin (10–15%), β-carotene (20–25%), and lutein (40–45%) (Walter and Strack 2011). Carotenoid accumulation in fruits begins throughout the ripening process alongside variations in pigmentation, sweetness, firmness, aroma, and acidity. Fruits contain different amounts of carotenoids depending on the species. Ripe tomatoes contain red lycopene, orange fruits include violaxanthin, and pepper fruits contain capsanthin. They are unique to their species and can all build up as an intermediate in the process (Marty et al. 2005).

In *Prunus* fruits, the accumulation of carotenoids is tightly regulated by various factors, including genetics, developmental stage, and environmental conditions. Transcription factors, such as members of the MYB and bHLH families, have been shown to control the expression of important carotenogenic genes. Light exposure, temperature, and nutrient availability also influence carotenoid biosynthesis and accumulation. Understanding these regulatory mechanisms is essential for developing strategies to enhance fruit carotenoid content and optimize agricultural practices. Researchers can use modern biotechnological techniques like genetic engineering to alter carotenoid biosynthesis pathways to achieve desired fruit coloration and nutritional profiles. Horticulturists have highlighted the importance of increasing carotenoid accumulation to produce visually appealing and nutritionally rich cultivars. This review examines recent developments in various carotenoid-related areas in *Prunus* species, including genes involved in regulatory mechanisms and key carotenoid biosynthesis profiles. The information gathered in this review elucidates the regulatory process governing the accumulation of carotenoids in *Prunus* fruits.

### Carotenoids: a colorful realm of *Prunus* species

#### Peaches

**Fig. 2:**
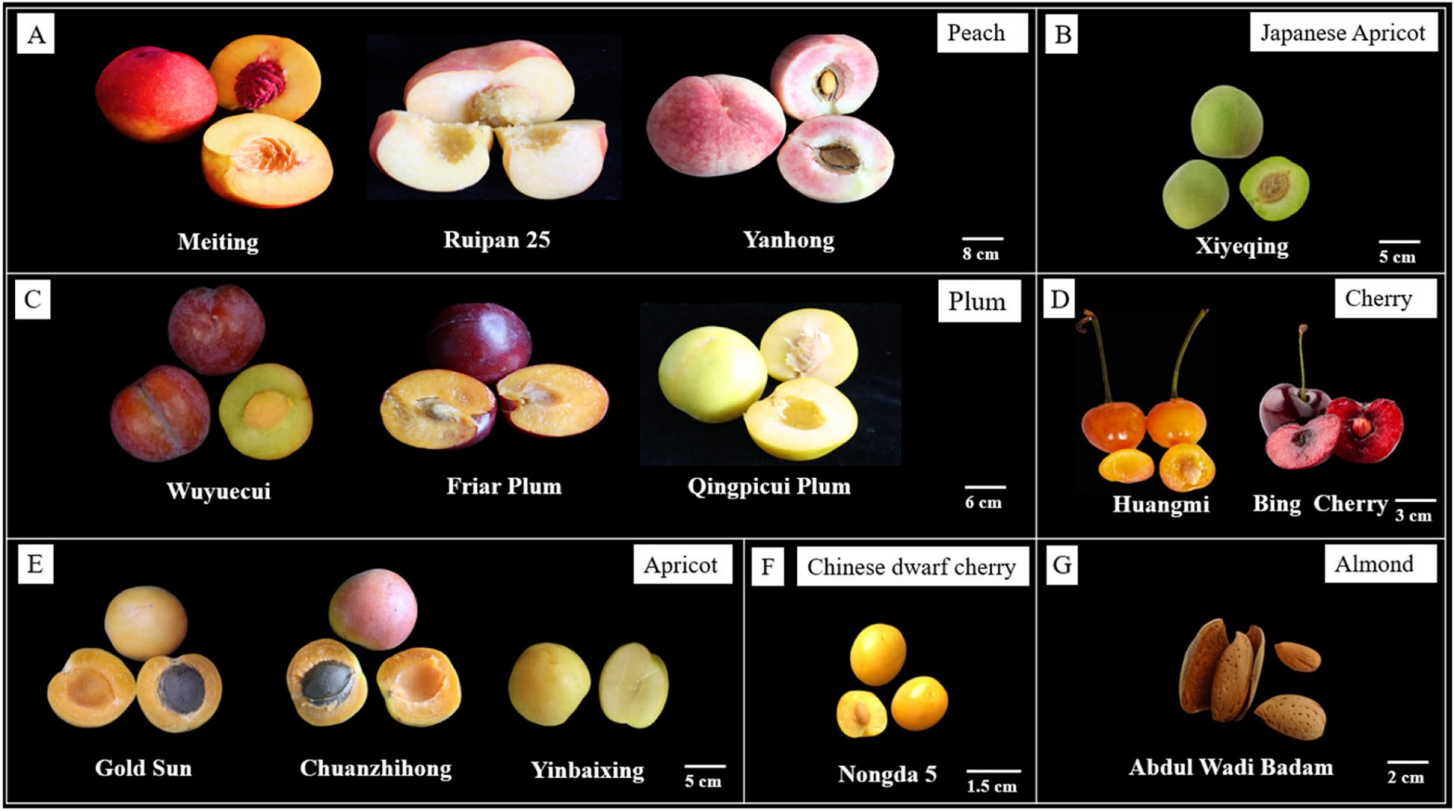
Diverse *Prunus* fruits **A** Various peach varieties; **B** Japanese apricot (“Gao, Z.H. (Ed.). (2024) (provided by Prof. Gao Zhihong, Nanjing Agricultural University); **C** Various plum varieties, Wuyuecui was provided by Prof. Liu Chaoyang, South China Agricultural University; **D** Cherries; **E** Various apricot varieties; **F** Chinese dwarf cherry (provided by Prof. Zhang Jiancheng, Shanxi Agricultural University); **G** Almond (provided by Iftikhar Alam, University of Kassel, Germany)

Peach (*Prunus persica*) is the most important commercial fruit of the *Prunus* species and the *Rosaceae* family. Peaches are valued for their aesthetic, nutritional, and organoleptic qualities. Carotenoids, sugars, acids, and volatile organic compounds (VOCs) all have a role in these qualities, and their levels vary depending on genetic, developmental, and post-harvest variables (Brandi et al. 2011). Since fruit carotenoid profiles are less diverse, genetic exploitation and quality enhancement related to fruit carotenoid production in peaches are constrained (Yan et al. 2020). Based on carotenoid content, peach pulp can be divided into two types: yellow and white (Fig. 2). Yellow-fleshed peaches are rich in carotenoids and greatly enhance the nutritional and commodity value of peaches (Cao et al. 2017; Veerappan et al. 2021). Carotenoids are formed in the white-fleshed peach fruit but later degrade into colorless substances, giving them a white color (Ma et al. 2014). The primary factor driving carotenoid degradation in white-fleshed peaches is the activity of the carotenoid cleavage dioxygenase (CCD) enzyme, particularly CCD4 (Brandi et al. 2011; Falchi et al. 2013). In yellow-fleshed peaches, the accumulation of carotenoids is primarily due to the inactivity of the CCD4 enzyme. When CCD4 is inactive, carotenoids are not degraded, allowing them to accumulate in the fruit flesh. This inactivity can result from various mutations in the *PpCCD4* gene (Ma et al. 2014; Fan et al. 2020). The *PpCCD4* gene on chromosome 1 of the peach genome encodes the enzyme responsible for carotenoid degradation (Falchi et al. 2013). This active CCD4 enzyme cleaves carotenoids, preventing their accumulation and producing a white flesh color (Ma et al. 2014). A study showed lower carotenoid levels in the white peach cultivar'Hujing'compared to the yellow-fleshed'Jinli'. It found lower carotenoid levels in'Hujing'likely due to higher *PpCCD4* expression. In Jinli, carotenoid accumulation was linked to specific gene expressions in skin and pulp. Post-harvest blue light treatment increased carotenoid content in'Jinli'by enhancing the expression of synthesis-related genes (Cao et al. 2017). Cryptoxanthin and violaxanthin are prevalent in yellow color peach fruits (Wang et al. 2023a). The initial step in managing the accumulation of carotenoids involves overseeing the transcription of the biosynthetic gene *BCH1*, which is the primary precursor for carotenoid production during peach fruit ripening. It was discovered that PpFPPS, PpGGPPS, PpLCYB, and PpBCH transcriptional regulations are partially responsible for the carotenoid accumulation in fruit skin during fruit development in the ‘Jinli’ peach variety. Several studies have investigated carotenoid profiles in different peach cultivars. A study analyzes carotenoid content in 15 yellow-fleshed peach cultivars, finding significant variations in total carotenoid content and individual carotenoid compositions. However, in addition to the transcriptions of the four genes mentioned above, *PpPDS* may also have increased in the pulp, which would explain the accumulation (Cao et al. 2017). The expression of *PSY, CCD,* and *BCH* varied dramatically between the Tibetan peaches grown at low and high altitudes (Zheng et al. 2023a, b). Yellow peach fruits have high carotenoid concentration and are abundant in different types of carotenoids, especially xanthophylls (Yan et al. 2020). The most prevalent carotenoid in the peach accessions studied was determined to be violaxanthin or β-cryptoxanthin, which is the end product of the carotenoid biosynthesis pathway (Gasic et al. 2016).

#### Apricots

Apricot (*Prunus armeniaca* L.) is one of the most important fruits in the *Rosaceae* family, and they are consumed primarily because of its distinctive juiciness, color, and taste. Apricots have a radiant color that ranges from orange or yellow to light yellow and white. The fruits of some cultivars can have a crimson blush against the yellow or orange background (Xi et al. 2020). Previous studies have demonstrated that apricots'orange or yellow peel color is attributed to carotenoids. During ripening, the apricot's skin abruptly shifts from green to white, yellow, orange, pink, or red (Erdogan-Orhan and Kartal 2011; Hou et al. 2016; Bourvellec et al. 2018; Xi et al. 2020). Cultivars with orange peel color had the highest total carotenoid content, followed by the yellow cultivars, whereas the light-yellow cultivars'quantity of carotenoids decreased as the fruit ripened (Xi et al. 2020). Most of the carotenoids that apricots acquire are β-carotene and the colorless carotenoids phytoene and phyto-fluene (Gasic et al. 2016). The orange-colored cultivar ‘Goldrich’ essentially accumulates β-carotene and primarily builds up with two colorless carotenoids, phytoene and phyto-fluene. In contrast, the off-white-colored variety ‘Moniqui’ only accumulates phytoene and phyto-fluene (Bureau et al. 2001). The carotenoid contents of the two cultivars ‘Kuchebaixing’(white flesh) and ‘Shushangganxing’ (orange flesh) were noticeably different, with β-carotene and (E/Z)-phytoene serving as the primary metabolites responsible for the variation in the total carotenoid content during the mature and ripen stages of fruit development (Zhou et al. 2021). Therefore, apricot fruits'yellow and orange skin color results from carotenoids (Ruiz et al. 2008).

The balance between carotenoid biosynthesis and degradation controls the molecular mechanism of fruit color in apricots. The primary pigment that gives yellow apricot fruits their color is β-carotene, one of the several carotenoids that accumulate in these fruits. During the ripening process, the lycopene beta-cyclase (*LCYB*) gene is dramatically increased, which promotes the synthesis of β-carotene. This gene causes lycopene to be converted to β-carotene, which builds up in the fruit's peel and flesh (Jiang et al. 2019; García-Gómez et al. 2020a, b; Xi et al. 2020). Yellow apricots accumulate β-carotene due to active biosynthesis and reduced degradation. In contrast, white apricots exhibit high carotenoid degradation mediated by *NCED,* leading to low β-carotene content and white flesh (Jiang et al. 2019). Apricot fruit contains a rich amount of β-carotene. There are a minor number of additional carotenoids like lutein, γ-carotene, lycopene, and cryptoxanthin in apricot fruit (Kurz et al. 2008). Compared to wild-type fruit, cultivated apricot fruit has a much higher concentration of β-carotene (Zhou et al. 2020). According to the studies, phytoene (about 10%) and β-carotene (60%) comprise most of the carotenoid content in apricot fruit. Because of an extraordinary absorption of β-carotene, many plants have a yellow, orange, or red color (Yuan et al. 2015). Specific yellow apricot cultivars have higher expression levels of genes involved in carotenoid biosynthesis, including *PSY, PDS, ZDS,* and *LCYB*. This enhanced expression results in increased carotenoid production (García-Gómez et al. 2020a, b). Previous study results revealed that *PSY, ZEP, CYP97, NCED1, CCD1*, and *CCD4* are the primary genes responsible for the significant variations in the total carotenoid content (Jiang et al. 2019); Zhou et al. 2021). The high and reduced transcription levels of *DXS* and *BCH1* genes are linked to higher levels of β-carotene accumulation in apricot (Yan et al. 2020).

#### Plums

Plum (*Prunus domestica* L.) is one of the most popular fruits in temperate regions, closely resembling apricots and peaches. Fruit color is a crucial characteristic that affects plum fruit quality. Plum contains numerous bioactive components, including carotenoids, that attract consumers because of their appealing color and nutritional value (Birwal et al. 2017). The carotenoid biosynthesis pathway in plum fruits begins with the conversion of precursor molecules into various carotenoids through several key enzymes. The process initiates with the synthesis of phytoene, facilitated by phytoene synthase (PSY). Subsequently, lycopene is produced through a sequence of desaturation and isomerization reactions, which further undergoes cyclization to form carotenes such as β-carotene (Kaulmann et al. 2016). In plums, the three main carotenoids found are β-carotene, lutein, and violaxanthin, serving as the most prevalent carotenoid in most of them (Gasic et al. 2016). Frier plum contains low carotenoid levels and minimal amounts of violaxanthin (Yan et al. 2020). In ripe fruits, color change is the essential phase in initiating carotenoid biosynthesis. Some genes, including *PSY, LCYB,* and *LCYE,* had distinct expression patterns during the carotenoid biosynthesis process (Chen et al. 2022a, b). However, β-carotene and carotenoids are not the main factors in the variation in the color of plum fruits (Lin et al. 2023). In a recent study, the carotenoid profiles and biosynthetic gene expressions among ten plum cultivars were comprehensively analyzed. The research reveals that the total and individual carotenoid contents were significantly higher in the skin compared to the flesh of the plums. Major carotenoids identified included lutein, zeaxanthin, β-cryptoxanthin, α-carotene, and β-carotene, with lutein and β-carotene being predominant in the skin and flesh, respectively (Deng et al. 2023).

The study also finds that the expression levels of key carotenoid biosynthetic genes, such as *PSY*, *LCYB*, and *LCYE,* positively correlated with the carotenoid contents (Sathasivam et al. 2021; Ampomah-Dwamena et al. 2022; Deng et al. 2023). Among these, the *PSY* plays a crucial role as the rate-limiting enzyme introducing carotenoid biosynthesis (Zhou et al. 2022; Deng et al. 2023). In contrast, *LCYB* and *LCYE* are essential for leading the synthesis of β-carotene and lutein, causing yellow and orange pigmentation, respectively (Deng et al. 2023; Yan et al. 2023). On the other hand, the negative correlation observed with *PDS* and *CRTISO* may reveal that their roles are influenced by regulatory mechanisms, substrate competition, or feedback loops, making them less central to carotenoid accumulation (Deng et al. 2023). Thus, *PSY, LCYB,* and *LCYE* emerge as the most important genes for carotenoid accumulation.

#### Sweet cherry

Sweet cherry (*Prunus avium* L.) has a pleasing appearance, tasty flavor, dietary benefits, and many biological functions. The presence of certain pigments, such as anthocyanins and carotenoids, causes the sweet cherry fruit to range in color from dark red to yellow (Wang et al. 2023a). Previous studies showed that the cherry has slightly superior carotenoids than apples, pears, and strawberries (Caller and Mackinney 1965). There doesn't seem to be any new phytoene synthesis during ripening, except for the cherry. Although the total amounts of carotenoid in the two cherry fruits were based on red and yellow varieties, results showed that compared to red fruits, the carotenoid concentration of yellow fruits appeared more constant after maturity (Chen et al. 2022a, b; Wang et al. 2023b). Every structural gene involved in carotenoid biosynthesis displayed positive associations between both varieties, except *ZEP* (Wang et al. 2023b). There is very limited knowledge about the regulatory mechanism of carotenoid biosynthesis in sweet cherries, and need to investigate more about cherry fruit.

#### Chinese dwarf cherry

*Prunus humilis* Bunge, also known as Chinese dwarf cherry, is a small perennial deciduous plant originating in northern China. Its fruit is also known as calcium fruit because of the high amount of calcium in it. Comparative genomic research found that *Prunus humilis* features are closely related to various *Prunus* fruit trees, including peach and apricot (Cheng et al. 2023; Zhang et al. 2014). Chinese dwarf cherry contains various kinds of carotenoids, although there is limited information about its carotenoid biosynthesis mechanism. The fruits also contain significant amounts of anthocyanins, flavanols, tannins, and other compounds and are rich in minerals, vitamins, organic acids, and amino acids (Cheng et al. 2023; Fu et al. 2020). The production of carotenoids in *Prunus* fruits requires further investigation, as limited research has been conducted despite their importance in the market.

#### Japanese apricot

The Japanese apricot (*Prunus mume* L.) is a woody fruit tree grown only in southern regions since it cannot tolerate cold winters and early springs. The Japanese apricot is typically collected green and unripe for its nutritional benefits before being processed commercially as a pickle or liquor (Inaba and Nakamura 1981; Kita et al. 2007; Wang et al. 2019). As a typical climacteric fruit, Japanese apricots lose their commercial value during processing as the surface color turns yellow because of the excessive ethylene production (Inaba and Nakamura 1981; Wang et al. 2019). In two Japanese apricot cultivars,'Orihime'and'Nanko', it was observed that'Nanko'had lower amounts of β-carotene and β-cryptoxanthin than'Orihime'. Furthermore, there may be a metabolic shift from α-carotenoid synthesis to β-carotenoid synthesis in Japanese apricot cultivars, marked by decreased *PmLCYE* expression and increased *PmLCYB* expression. During fruit ripening, the expression levels of seven potential carotenogenesis genes involved in producing carotenoids, such as β-carotene, lutein, violaxanthin, and zeaxanthin, change significantly. Consequently, among all the downstream carotenogenic genes, the upregulation of *PmLCYB* likely plays a crucial role in carotenoid accumulation in Japanese apricot fruits (Kita et al. 2007). Thus, *PmLCYB* may play a pivotal role in the β-carotenoid accumulation as well as in massive carotenoid accumulation caused by downstream carotenogenic genes in Japanese apricots.

#### Almond

Almond (*Prunus dulcis* L.), belonging to the Rosaceae family, is a stone fruit utilized in the food industry and is important in the agricultural sector and the economy. It was found that the immature almond fruits of the various varieties produce large amounts of linalool and geraniol, two terpene volatiles. Almonds are more renowned for their content of healthy fats, proteins, fiber, and various essential nutrients (Nawade et al. 2019). However, almonds do contain small amounts of specific carotenoids. For example, β-carotene and lutein are present in almonds, though not in concentrations as high as in other *Prunus* fruits more traditionally associated with carotenoid content (Stuetz et al. 2017). These carotenoids are antioxidants and have potential health benefits, contributing to overall well-being.

In summary, among *Prunus* species, the carotenoid biosynthesis pathway is quite similar to other plants. Phytoene synthase (PSY), phytoene desaturase (PDS), zeta-carotene desaturase (ZDS), lycopene beta-cyclase (LCYB), and lycopene epsilon-cyclase (LCYE) are key enzymes that are present among all species, which help in the production of carotenoids including β-carotene, lutein, zeaxanthin, and violaxanthin (Kita et al. 2007; García-Gómez et al. 2020a, b; Lin et al. 2023). Similar transcription factors, such as AP2/ERF and bHLH, control these enzymes, which regulate the genes involved (Liang and Li 2023). Despite these common characteristics, Carotenoid types and their concentrations vary significantly among *Prunus* fruits. Peaches are rich in beta-cryptoxanthin and β-carotene, giving them their orange hue (Burri et al. 2016), whereas apricots mainly accumulate β-carotene (Jiang et al. 2019).

On the other hand, Plums and cherries have lower levels of carotenoids, with lutein and zeaxanthin being more common (Kaulmann et al. 2014). In cherries, anthocyanins frequently conquer the carotenoids, contributing to their dark red color (Gonçalves et al. 2021). These variations are relatively due to gene expression and replication differences amongst *Prunus* species. Moreover, the carotenoids in plastids vary among species, with some having more efficient storage structures like plastoglobules (Yan et al. 2020). Environmental factors, such as light and temperature, further form the carotenoid profiles by affecting the pathway in species-specific ways.

These unique characters offer thrilling prospects for further research. Comparative studies will help uncover how carotenoid biosynthesis genes evolved in *Prunus*. In contrast, functional studies like gene transformations in these species can shed light on the genetic and biochemical mechanisms behind the diversity of carotenoids in *Prunus* fruits.

### Carotenoids and* Prunus* species: their importance for humans

*Prunus* fruits such as peaches, apricots, and plums are popular for their delicious essences and assist as substantial sources of carotenoids, which are vital for human nutrition and contribute to the fruits'visual appeal, as shown in (Fig. 2). Carotenoids play an essential role in our health by converting into vitamin A, which is vital for many human body functions. Unfortunately, vitamin A deficiency continues to be a serious problem, especially in developing countries with limited access to nutrient-rich foods (Meléndez-Martínez 2019). Different types of carotenoids are found in our diets; some are lipid-dissolved, like those found in dairy products and egg yolks, while others exist in crystalline forms, such as in carrots, tomatoes, peaches, and apricots. The way carotenoids are deposited in these foods affects how easily our bodies can absorb them during digestion. Carotenoids help lower the risk of chronic diseases, such as cancer, and promote eye health (Schweiggert and Carle 2017).

Xanthophylls in mature fruits such as peaches, plums, and apricots tend to be in the esterified form. Carotenoid esters, particularly dominant in these fruits, boost savor and improve the bioavailability of carotenoids (IIahy et al. 2019; Yan et al. 2020). These esters form when xanthophylls like lutein, cryptoxanthin, and zeaxanthin bind with fatty acids, increasing their stability and solubility (Mercadante et al. 2017). During digestion, these esters are hydrolyzed, allowing carotenoids to be absorbed effectively, often with a bioavailability that rivals or exceeds free carotenoids. This enhanced absorption ensures that carotenoids can fulfill their biological roles, acting as antioxidants and supporting human health (Petry and Mercadante 2019). The lively colors of *Prunus* fruits, driven by their carotenoid content, are key sensory traits that attract consumers and enhance market value.

The health benefits related to carotenoids are extensive. They are well-known for their antioxidant properties, helping to reduce the risk of chronic diseases such as age-related macular degeneration, cataracts, cardiovascular diseases, and certain types of cancer (Eggersdorfer and Wyss 2018). Carotenoids also play a role in supporting immune function and reproductive health. β-carotene acts as a pro-vitamin A source, crucial for maintaining good vision and skin health (Von Lintig 2010). Plums are particularly rich in bioactive compounds like carotenoids, which help to fight against oxidative damage and promote general well-being (Birwal et al. 2017; Zheng et al. 2020). Likewise, sweet cherries and apricots exhibit high antioxidant activities that help maintain mitochondrial function and protect cellular structures from oxidative stress (Alvarez-Suarez et al. 2017).

Beyond their health benefits, carotenoids have applications in cosmetics due to their protective properties for the skin. Some *Prunus* species, such as *Prunus humilis* Bunge, offer unique nutritional advantages like high calcium content (Fu et al. 2020). The multifaceted roles of carotenoids, from enhancing fruit quality and consumer appeal to improving human health, highlight their significance in nutrition and commercial applications. Future research to increase carotenoid levels in *Prunus* fruits could further enhance their health benefits and market potential, solidifying their status as functional foods.

### Carotenoid sequestration and its diversity in fruits of *Prunus* species

Chromoplasts are a unique form of plastid that play a key role in producing and storing carotenoids in plants. Plastid’s internal arrangements can facilitate carotenoid sequestration by improving the chemical stability of carotenoid molecules. For instance, developing plastoglobules is an essential plastid arrangement event that can provide carotenoids to a stable, photo-degradation sequestered site (Merzlyak and Solovchenko 2002; Yan et al. 2020). Fruits are sink organs that primarily store carbohydrates and coloring pigment “carotenoids”, which build up in large amounts in chromoplasts in forms resembling crystals or plastoglobuli​. The development and differentiation of plastids, particularly chromoplasts and plastoglobules, are regulated by specific genes such as *OR, fibrillin, PDV,* and *ARC*. The *OR* (orange) gene promotes chromoplast differentiation and carotenoid accumulation, while fibrillin maintains a plastid structure and facilitates plastoglobule formation. *PDV* genes ensure proper plastid division, and *ARC* genes regulate chloroplast size and number. These genetic mechanisms are crucial for optimizing carotenoid content in plants (Yan et al. 2020).

**Fig. 3:**
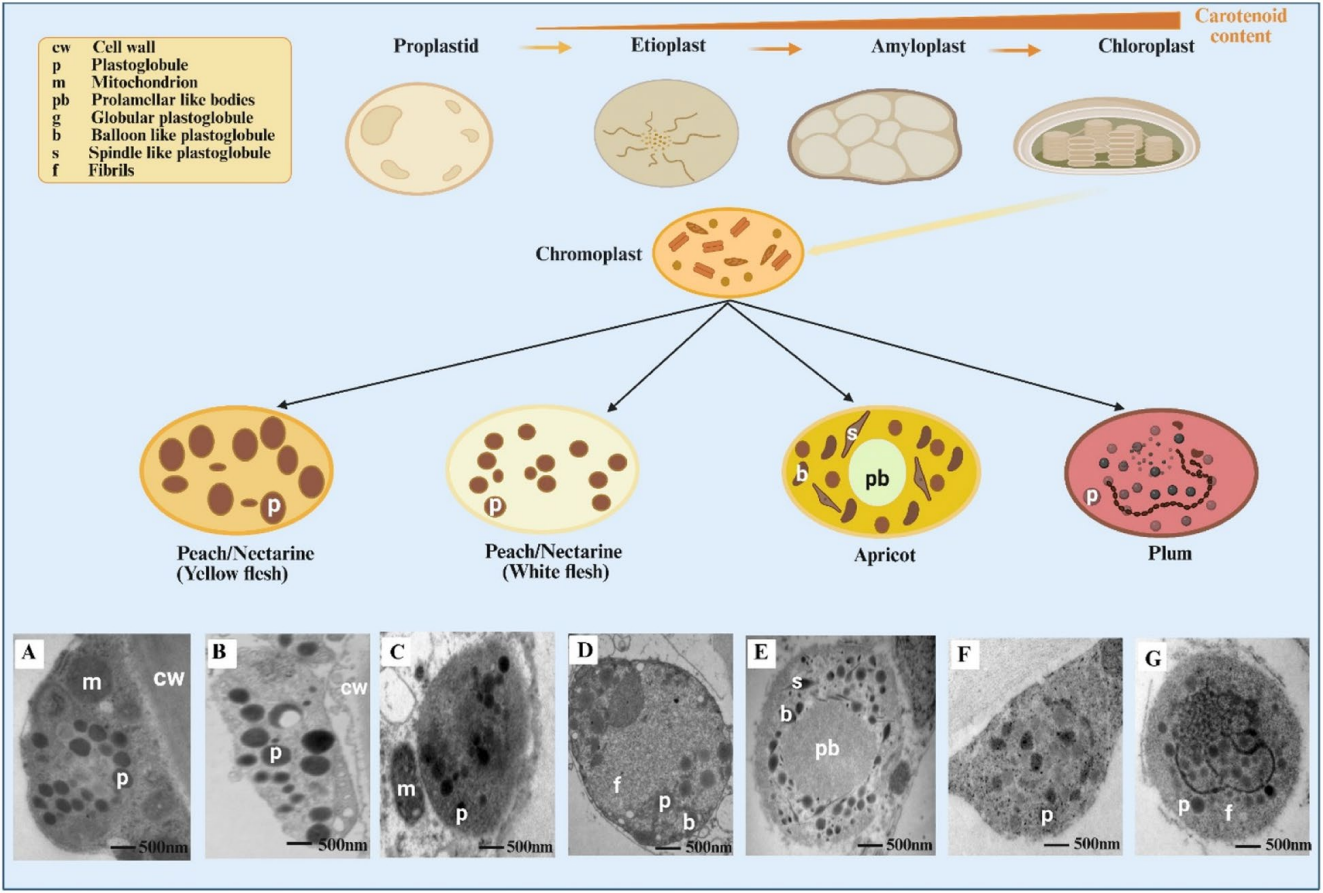
Pro-plastids are the ancestors of all other plastid types. An Etioplastic is a poorly formed plastid with little carotenoid production activity. Amyloplasts are plastids that contain starch and have a low to moderate range of carotenoid accumulation. Chloroplasts are characteristic of plants where carotenoids and chlorophyll are synthesized coordinately. A chromoplast is a plastid that accumulates carotenoids and contributes to the vibrant colors observed in various plant organs. Various *Prunus* fruits contain different sizes, plastoglobuli structures, and densities. **A**– **G**, TEM micrographs of plastids (**A**)Yellow Nectarine (**B**) Yellow Peach (**C**) White Nectarine (**D**) White Peach (**E**) Apricot (**F**) Plum (Yan et al. 2020) (**G**) Plum (orange flesh)

Furthermore, the bio-accessibility and bio-efficacy of carotenoids are influenced by factors including food matrix, processing methods, digestion efficiency, chemical structure, nutrient interactions, and individual genetic differences (Priyadarshani 2017). Understanding both the genetic regulation of plastid development and the factors affecting carotenoid bioavailability is essential for enhancing carotenoid-rich foods'nutritional quality and health benefits. Transmission electron microscopy (TEM) assay revealed that *Prunus* fruits have a shared globular chromoplast. On the other hand, plastid and plastoglobuli size and numbers vary between species, particularly in the case of white-flesh Ruiguang-19 nectarine, having plastids comparable to chromoplasts but smaller plastoglobules. Studies revealed that various shapes of plastoglobuli are found in apricots, such as globular, balloon, and spindle-shaped. In the chromoplast of apricots, prolamellar bodies are also found, as shown in (Fig. 3). Furthermore, Friar plums have plastid-related genes with the lowest transcription level, and it was assumed that *OR* and *GLK2* genes were linked to the largest chromoplasts present in apricots (Yan et al. 2020). Although prior investigations showed that the mature flesh of apricot, peach, and plum fruits accumulate carotenoids, a comprehensive comparison of their various carotenoid profiles is lacking from these studies (Fratianni et al. 2013; Yan et al. 2020; Sun et al. 2022). Investigating the structural behavior of plastids that cannot store carotenoids but have effective carotenoid biosynthesis regulation is essential. The carotenoid regulation in fruits can be moderately complex because of the ripening process’s drastic changes in content and composition, which also depend on the fruit tissue and stage of development. Carotenoid accumulation balances its synthesis and degradation. Carotenoid accumulation is influenced by cytological variables such as plastid growth and carotenoid sequestering structures. The regulation of carotenoid biosynthesis is interconnected with related metabolic pathways, plastid differentiation, and developmental and environmental responses in which these isoprenoid pigments are involved (Zheng et al. 2019; Zacarías-García et al. 2021). The transcriptional control of carotenogenic genes does not always reflect the diversity of carotenoid accumulation (Lado et al. 2015a; Zhu et al. 2018). The relationships between plastid features, gene transcription, and carotenoid accumulation were discovered that the development of chromoplasts is probably more significant in influencing carotenoid accumulation than carotenogenic transcription in *Prunus* fruits (Yan et al. 2020). As a fruit ripens, the chloroplast and chromoplast synthesis capacities increase, leading to carotenoid accumulation, as shown in (Fig. 3).

### Comparison of carotenoid profiles among various *Prunus* species

As a plant develops, chromoplasts can be transformed from chloroplasts. For carotenoid sequestration during ripening, the plastids in fruits must all convert from chloroplast to chromoplast (Lado et al. 2015b). This conversion mechanism has repeatedly been demonstrated to result in different carotenoid profiles among species rather than a conservative carotenoid profile (Howitt and Pogson 2006). Many *Prunus* fruits, including apricots, peaches, and plums, are rich in carotenoids as they ripen. *Prunus* species vary widely in their carotenoid composition; yellow-fleshed peaches and yellow-fleshed apricots are typical examples of the genus *Prunus* with rich carotenoids in fruits (Yan et al. 2020; Wang et al. 2023b). Fruits like peaches and apricots have notable variations in carotenoid levels and characteristics (Wang et al. 2023b). According to HPLC-PAD analysis, the ripe fruits of *Prunus* species accumulate carotenoid esters; however, the concentration and profiles vary significantly (Yan et al. 2020). Numerous studies have shown that both carotenogenic genes and gene regulation in plants are responsible for the carotenoid profiles and levels in plants (McQuinn et al. 2015; Wisutiamonkul et al. 2017; Ma et al. 2018). The promoter plays a crucial role in controlling gene expression, determining the variety in carotenoid concentration and composition. The transcriptomic study revealed a strong correlation between *BCH1* expression levels and the carotenoid profiles of peach and apricot fruit (Wang et al. 2023a). Although the mature flesh of plum, apricot, cherry, peach, and nectarine fruits all contain carotenoids, as shown in (Table 1). Studying carotenoid biosynthesis in *Prunus* species provides valuable insights into the complex molecular processes underlying fruit coloration, nutritional content, and horticultural significance. By sorting out the complexities of carotenoid metabolism and regulation, researchers pave the way for developing improved fruit cultivars with enhanced visual appeal and health benefits. Plant carotenoid profiles have consistently been correlated with the expression patterns of genes involved in carotenoid biosynthesis.

**Table 1:** Diversity of main carotenoids found in *Prunus* species

Species	Lutein	Zeaxanthin	β-Cryptoxanthin	α-Carotene	β-Carotene	Total Carotenoids Contents (mg\kg FW)	References
Yellow Apricot	0.09–0.98	0.03–0.49	2.11–9.78	2.31–5.79	19.71–33.74	22.94–49.07	(Ruiz et al. 2005; Zhang et al. 2019a, b; Zhou et al. 2020)
White Apricot	0.043–0.58	0.03–0.06	0.17–1.40	0.02–0.42	1.50–13.00	4.48–25.12	(Ruiz et al. 2005; Zaghdoudi et al. 2015; Zhou et al. 2020)
Yellow Peach/Nectarine	0.25–0.27	1.10–2.25	0.42–0.51	0.43–0.45	4.35–8.19	6.45–11.17	(Gil et al. 2002; Brandi et al. 2011; Wen et al. 2020; Yan et al. 2020; Song et al. 2022)
White Peach/Nectarine	0.5–9.35	-	0.10–0.12	-	0.04–0.08	1.23–10.27	(Gil et al. 2002; Zaghdoudi et al. 2015; Wen et al. 2020)
Sweet cherry	2.4–2.7	1.1–2.1	-	1.6–3.1	1.6–2.9	3.56–10.78	(Demir 2013; Średnicka-Tober et al. 2019)
Plum	1.61–17.62	0.68–2.97	0.03–0.13	0.8–2.83	0.40–18.80	0.4–1.88	(Gil et al. 2002; Díaz‐Mula et al. 2008; Yan et al. 2020)
Chinese dwarf Cherry	-	-	-	-	-	10–28	(Cheng et al. 2023; Yang et al. 2023)
Japanese Apricot	1–2.2	0.2–0.6	0.3–0.9	1–7	0.5–6.5	0.03 −0.12	(Kita et al. 2007; Oe et al. 2012)
Almond	0.84–0.96	00.1–00.3	-	-	00.1–00.12	0.07–0.58	(Stuetz et al. 2017; El et al. 2024)

The carotenoid profile is for all *Prunus* species in the flesh except almonds (seed).—represents that carotenoids were absent or present only in trace amounts

Analysis of fruit carotenoid profiles of a large number of *Prunus* species showed that peaches and plums are also noticeably different from those of apricots (Ruiz et al. 2005; Song et al. 2011; Demir et al. 2013; Zaghdoudi et al. 2015; Yan et al. 2020; Zhou et al. 2020; Song et al. 2022), which primarily accumulate phytoene, phyto-fluene, and β-carotene. Previous studies revealed that white-fleshed varieties typically have lower carotenoid levels, although some exceptions with higher carotenoid contents exist (Ruiz et al. 2005; Zaghdoudi et al. 2015; Zhou et al. 2020), as shown in (Table 1). Further investigation is needed to understand the underlying mechanisms responsible for this variation. Cherries primarily accumulate β-carotene, lutein, and zeaxanthin, but their carotenoid levels are often dominated by anthocyanins (Ferretti et al. 2010; Demir 2013; Kapoor et al. 2022). Japanese apricots contain significant amounts of β-carotene, lutein, and neoxanthin, with variability depending on the cultivar and ripening stage (Ding et al. 2023b). Although comprehensive information about carotenoids for *Prunus humilis* is little, it is predictable to mirror apricots with β-carotene, lutein, and minor xanthophylls (Yang et al. 2023). Almonds, on the other hand, have low carotenoid content, with trace amounts of β-carotene and lutein, as their kernels lack fleshy tissue (El et al. 2024).

Overall, the carotenoid composition varies significantly across these species, influenced by their unique genetic and metabolic traits. The key phenotypes include the intensity and distribution of red, orange, or yellow pigmentation in the fruit skin and flesh (Ranganath 2022). The coloration is often used as an indicator of carotenoid content. Carotenoid content plays a significant role in influencing the size and shape of *Prunus* fruits (Nowicka et al. 2023). Some varieties may exhibit larger or more rounded fruits due to higher carotenoid accumulation, which can be a desirable trait (Zhang et al. 2019a, b). A phenotype to observe is the depth and consistency of coloration within the fruit's flesh. Carotenoids contribute to the flavor and aroma of *Prunus* fruits. Ripen fruits with high carotenoid content often have a sweeter and more aromatic profile (Vicente et al. 2011). Sensory evaluations can be used to assess these traits. Texture attributes may include firmness, crispness, or softness. Carotenoid-rich fruits can exhibit differences in texture and juiciness, which are important for consumer preferences and marketability (García-Gómez et al. 2020a, b). Phenotypic evaluation of carotenoid traits in *Prunus* fruits plays a crucial role in breeding programs, quality assessment, and the development of fruit varieties with improved carotenoid content and associated attributes.

Numerous *Prunus* species have a wide range of carotenoid profiles and plastid configurations. Surprisingly, mature fruits from the *Prunus* species under study displayed unique carotenoid profiles and concentrations, indicating greater diversity than the peach or apricot group. Understanding the molecular mechanisms underlying carotenoid synthesis and diversity in *Prunus*, Research on the transcripts involved in plastic development and carotenogenesis was associated with carotenoid profiling. A Transcriptional study reveals that variations in carotenoid content among the *Prunus* species (peach, apricot, and plum) are influenced by differences in gene expression associated with carotenogenesis, carotenoid sequestration structures, and carotenoid degradation pathways. Peaches and apricots exhibit higher carotenoid biosynthesis and effective sequestration except for the Ruiguang-19 nectarine variety, while plums show reduced biosynthesis and higher degradation activity (Yan et al. 2020). The difference in carotenoid levels between these *Prunus* fruits is not solely due to DEGs. Still, it is also influenced by functional aspects such as enzyme activity, regulatory mechanisms, and the structural ability to store carotenoids. The extent of these factors varies across cultivars, environmental conditions, and developmental stages. Further research is needed to confirm whether this trend is consistent across other cultivars of different *Prunus* species.

### Upstream regulators and environmental drivers in carotenoid biosynthesis

**Fig. 4:**
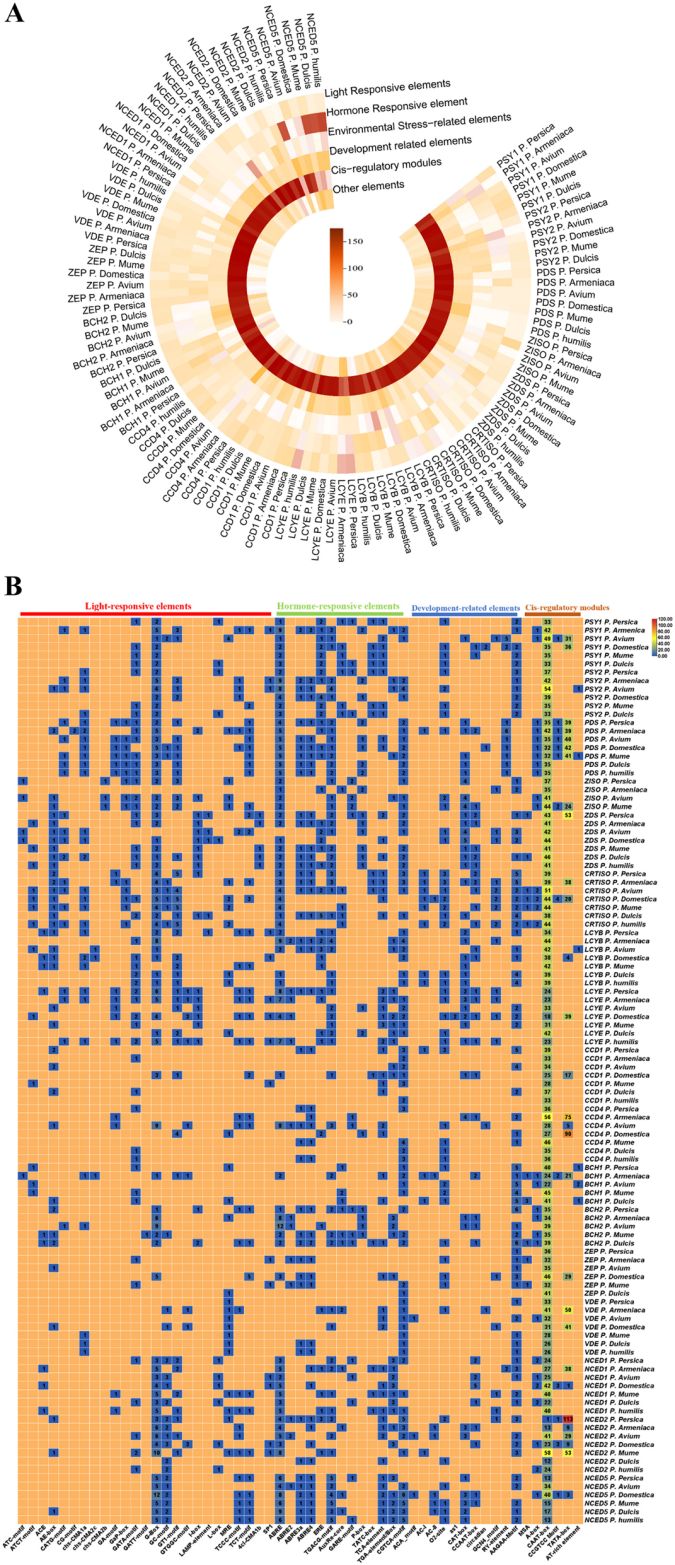
Impact of upstream regulators on carotenoid biosynthesis in *Prunus* species. **A** Comparative analysis of *cis*-regulatory elements in the crucial genes of the carotenoid biosynthesis pathway in various *Prunus* species. **B** Heatmap showing the distribution of *cis*-element families in essential genes of carotenoid accumulation in *Prunus* species. This indicates that *Prunus* species simultaneously respond to multiple *cis*-elements and environmental stress factors

*PSY, ZEP, BCH, NCED,* and *CCDs* are the most significant genes responsible for carotenoid biosynthesis in *Prunus* species (Deng et al. 2023). These genes are integral to the carotenoid biosynthetic pathway, each contributing to different steps and regulatory points determining the Prunus species'composition and levels of carotenoids. Their coordinated expression and regulation are essential for the fruits'proper development, coloration, and stress responses. The major genes responsible for the significant variations in the total carotenoid concentration are *PSY, LCYB*, and *BCH*. The genes associated with carotenoid biosynthesis are listed with their accession numbers in (Table 2) for various *Prunus* species. The regulation of carotenoid biosynthesis in *Prunus* involves the integration of light signaling, hormonal responses, and transcriptional regulation. This complex network ensures that carotenoid levels are optimized according to the fruit's physiological and developmental requirements, affecting its color, nutritional content, and market quality. New transcription factors regulating gene expression in carotenoid production have been identified as molecular and bioinformatic techniques used in *Prunus* fruits and are now more widely available. Understanding these regulatory properties will eventually aid biotechnological plant modification methods. Genomic data facilitate the understanding of carotenoid synthesis and regulation gene information in *Prunus*, for example, through an extensive *cis*-element analysis on 17 genes associated with the carotenoid biosynthesis pathway across seven *Prunus* species: *Prunus persica, Prunus humilis, Prunus mume, Prunus avium, Prunus armeniaca, Prunus dulcis,* and *Prunus domestica*. The sequence data from these different *Prunus* species provide insights into the regulatory mechanisms influencing carotenoid biosynthesis (Fig. 4A). The comprehensive analysis of *cis*-acting regulatory elements in carotenoid biosynthesis genes across *Prunus* species reveals the complex and species-specific regulatory mechanisms leading to carotenoid accumulation. Light-responsive elements, particularly G-box motifs, are key in enhancing gene expression influenced by light exposure and circadian rhythms. Hormone-responsive elements, such as those related to ABA and ethylene, integrate environmental stress signals like drought and salinity into the biosynthetic pathways, whereas stress-related elements, including those associated with *CCD* genes, contribute to abiotic stress adaptation. Development-related elements, regulated by transcription factors such as NAC and WRKY, further regulate carotenoid accumulation during fruit ripening. This active regulatory network, responsive to environmental factors like temperature and light, provides crucial understanding about developing targeted strategies to improve fruit quality and stress resilience in *Prunus* species.

**Table 2:** The genes of the carotenoid biosynthesis pathway from the seven *Prunus* species

**Genes**	**Arabidopsis**	**Apricot**	**Peach**	**Cherry**	**Plum**	**Japanese Apricot**	**Chinese dwarf cherry**	**Almond**
PSY1	AT5G17230	AY822067.1	XM_020560648.1	PAV_17813g00020.1	Pd.00g731680.m01	AB253628.1	CH0226773.1	Prudul26A025588T2
PSY2	-	MH412664.1	XM_007215345.2	XM_021968140.1	Pd.00g025960.m01	XM_008229666.2	CH0226774.1	XM_034351273.1
PDS	At4g14210	AY822065.1	KJ789375.1	PAV_9518g00010.1	Pd.00g912910.m01	XM_008224486.1	CH0204021.1	XM_034370405.1
ZISO	AT1G10830	MK117280.1	KM399420.1	XM_021945486.1	-	XM_008246202.1	-	AP019297.1
ZDS	At3g04870	MK117279.1	KJ789376.1	XM_021961825.1	Pd.00g1017090.m01	XM_008219979.2	CH0206291.1	XM_034366565.1
CRTISO	AT1G06820	PARG26577m01	XM_007220597.2	XM_021949174.1	Pd.00g932250.m01	XM_008242896.1	CH0224084.1	XM_034370676.1
LCYB	AF117256.1	MK117277.1	XM_007201984.2	XM_021974374.1	Pd.00g860120.m01	XM_008242507.1	KR866276.1	XM_034368113.1
LCYE	AT5G57030	MK117278.1	XM_007203578.2	XM_021949344.1	Pd.00g192930.m01	XM_008244160	CH0222845.1	Prudul26A015290T1
CCD1	AT3G63520	MH824416.1	XM_007220597.2	XM_021965066.1	Pd.00g797400.m01	XM_008233031.2	CH0212815.1	XM_034347354.1
CCD4	AT4G19170	PARG06146m01	XM_007222371.2	XM_021954085.1	Pd.00g989950.m01	XM_008223563.1	CH0203116.1	XM_034344322.1
BCH1	AT4G25700	-	XM_007211073.2	XM_021965165.1	-	XM_008240722.2	CH0213722.1	XM_034360217.1
BCH2	At5g52570	XM_007218653	XM_020568658.1	XM_021971362.1	-	XM_008236616.2	CH0221851.1	XM_034344122.1
ZEP	AT5G67030	AF071888.1	XM_020568194.1	XM_021969154.1	Pd.00g713100.m01	AB253634.1	CH0223599.1	XM_034367922.1
VDE	AT1G08550	PARG03208m02	XM_020567342.1	XM_021945104.1	Pd.00g1018650.m01	AB298289.1	CH0205955.1	XM_034366379.1
NCED1	AT3G63520	PARG11107m01	XM_007214595.2	XM_021976779.1	Pd.00g167140.m01	XM_008228017.1	CH0214553.1	XM_034356767.1
NCED2	AT4G18350	PARG11892m01	EU912386.1	FJ560910.1	Pd.00g093690.m01	-	CH0214554.1	XM_034357553.1
NCED5	AT1G30100	OZ177840.1	XM_020562736.1	XM_021978488.1	Pd.00g640080.m01	XM_008227273.1	CH0215198.1	Prudul26A007836T1

The *cis*-acting elements contained in the gene promoter sequence can be predicted through some special tools, such as the online website Plant CARE database (http://bioinformatics.psb.ugent.be/webtools/plantcare/html/). Interestingly, significant variations in the total number of *cis*-elements among different *Prunus* fruit species indicate potential differences in the mechanisms that regulate gene expression. Especially the abundance of *cis*-elements in *Prunus Armeniaca* (1551), followed by *Prunus Avium* (1375), *Prunus Domestica* (1477), *Prunus Persica* (1353), *Prunus Mume* (1346), *Prunus Dulcis* (1024), and *Prunus Humilis* (729) (Fig. 4B). All members of the *Prunus* species contain five different elements families like (1) light-responsive *cis*-elements such as 3-AF1, AT1-motif, ATC-motif, ATCT-motif, ACE, AE-box, CATG-motif, CG-motif, chs-CMA1a, chs-CMA2c, chs-CMA2b, chs-CMA1a, GA-motif, Gap-box, GATA-motif, GATT-motif, G-Box, GC-motif, GT1-motif, GTGGC-motif, I-box, L-box, MRE, TCCC-motif, TCT-motif, 4 cl-CMA1b, Sp1, and LAMP-elements. Light exposure is particularly crucial, as it directly affects the expression of key biosynthetic genes. Studies reveal that increasing light exposure increases the expression of genes linked to carotenogenicity, which raises the carotenoid content of *Prunus* fruits (Ohmiya et al. 2019). Light signaling and circadian rhythms are crucial in regulating carotenoid biosynthesis in *Prunus* species, affecting pigmentation and fruit quality. Light quality, intensity, and duration influence the expression of carotenoid biosynthesis genes, such as *PSY* and *PDS,* by interacting with light-responsive elements like the G-box in their promoters. Phytochromes are light-sensitive proteins and are crucial in signal transduction to the nucleus. They interact with phytochrome-interacting factors (PIFs), a transcription factor group that integrates environmental signals to regulate the expression of genes involved in carotenoid metabolism (Zhang et al. 2019a, b). PIF3 binds to these G-box motifs, adjusting gene expression in response to varying light conditions. The circadian rhythm further regulates the timing of gene expression, coordinating carotenoid biosynthesis with the most favorable light periods (Stanley and Yuan 2019). In regions with fluctuating daylight lengths, such as those with long or short light conditions, these mechanisms can greatly influence carotenoid accumulation, affecting fruit color and nutritional quality. For instance, red light treatments have been demonstrated to boost carotenoid content in citrus (Huang et al. 2022), indicating possible applications in *Prunus* cultivation to enhance fruit quality. (2) Hormone-responsive elements such as ABRE, ABRE2, ABRE3a, ABRE4, ERE, TGACG-motif, AuxRe-core, GARE-motif, P-box, TATC-box, TCA-element, CGTCA-motif, and TGA-elements. The ABRE, a crucial element in ABA-mediated stress responses, controls the expression of genes involved in Osmo-protectant synthesis and stress tolerance, essential for sustaining fruit production under abiotic stresses such as drought and salinity (Liu et al. 2023; Aziz et al. 2025). Drought, salinity, and excessive light can influence carotenoid biosynthesis, particularly affecting the expression of genes such as *PSY* (Kapoor et al. 2022). Light signals can enhance ethylene production, which promotes the expression of carotenogenic genes; This suggests a synergistic effect where light activates the biosynthesis pathway and modulates hormone levels that further influence gene expression (Zhang et al. 2019a, b; Wang et al. 2023a). (3) Environmental stress-related elements, i.e. LTR, MYC, MYB, ARE, S box, A- Box, W-box, MBS, as-1, and WUN-motifs. Water stress has been observed to affect carotenoid biosynthesis in *Prunus* species. The *carotenoid cleavage dioxygenase (CCD)* gene family is essential in the carotenoid biosynthesis pathway and is responsible for cleaving carotenoids into apocarotenoids, which are crucial for plant physiology, development, and stress tolerance. The *CCD* gene family is responsible for its phylogeny and expression patterns in the *Prunus* species. In *P. mume*, ten non-redundant *PmCCDs* (*CCD1, CCD4, CCD7, CCD8, NCED,* and *CCD*-like) have been identified and distributed across three chromosomes. These genes exhibit tissue-specific expression and are responsive to stress conditions like salt and drought, suggesting their involvement in stress resistance. *PmCCDs* display interspecific collinearity with other *Prunus* species, such as *P. armeniaca* and *P. persica*, indicating conserved functions across these species(Ding et al. 2023a). Understanding the expression and regulation of *CCDs* in *Prunus* can enhance fruit quality and stress tolerance, particularly in the context of global climate change. (4) Development-related elements include ACA-motif, AC-I, AC-II, O2-site, CAT-box, CCAAT-box, circadian, GCN4-motif, AAGAA-Motif, MSA, and RY-elements. NAC and WRKY transcription factors are known to regulate carotenoid-related genes during the process of fruit ripening in *Prunus* fruits (Zhang et al. 2019a, b). (5) Cis-regulatory Modules (CRMs) such as A-box, CAAT-box, CCGTCC Motif/box, TATA-box, and AT-rich element, this implies that most members of the *Prunus* species may respond to promoter *cis*-elements and environmental stress elements at the same time. This information emphasizes the complex regulatory environment governing carotenoid production in *Prunus* fruits, highlighting the critical role of *cis*-regulatory elements in shaping gene expression patterns across different species, thereby influencing growth and development. These results highlight the necessity of thoroughly understanding the specific regulatory mechanisms in each *Prunus* species to unravel the specifics of fruit color development. Temperature also plays a significant role in carotenoid accumulation. Research about apricots states that cooler temperatures during fruit development can result in higher carotenoid content, especially β-carotene (Ruiz et al. 2005). The *cis*-elements identified from the promoter sequences of *Prunus* species are associated with specific transcription factors (TFs) that regulate gene expression. Light-responsive elements such as the G-box, GATA-motif, I-box, and L-box interact with transcription factors like bHLH (e.g., PIF3, PIF4, and PIF5), which regulate photomorphogenesis and light signaling, and HY5 (Elongated Hypocotyl 5), a critical regulator of photoreception (Jing and Lin 2020). Circadian clock-related TFs like CCA1 and LHY further modulate these light-responsive genes, influencing pathways like carotenoid biosynthesis (Patitaki et al. 2022). Additionally, members of the MYB family and TCP TFs bind to these elements, contributing to light-dependent gene regulation (Viola and Gonzalez 2023). Hormone-responsive elements such as ABRE, ERE, and AuxRe-core are critical for responses to ABA, ethylene, and auxin (Duan and Schuler 2005). Key transcription factors include ABI5 and AREB/ABF for ABA signaling, EIN3 and ERF for ethylene responses, and ARFs (Auxin Response Factors) for auxin-regulated processes. Gibberellin-responsive motifs like the GARE motif are regulated by DELLA proteins and PIFs, integrating light and hormone signaling (Luo et al. 2023). Similarly, the TCA element involved in salicylic acid responses interacts with TGA TFs. Environmental stress-responsive elements such as the MYC and MYB binding sites ARE, and the W-box play significant roles in abiotic stress tolerance. MYC2 regulates drought and salinity responses, while MYB TFs like MYB96 mediate ABA-dependent stress adaptation (Zhang et al. 2019a, b). Development-related elements like the CAT-box and RY-element are regulated by TFs such as NACs (Li et al. 2022), modulate ripening and senescence, and seed-specific TFs like FUS3, ABI3, and LEC1(Verma et al. 2022). The CAAT-box and TATA-box, essential components of *cis*-regulatory modules (CRMs), facilitate the recruitment of general TFs like TBP and NF-Y to initiate transcription (Matuoka and Chen 2002).

Transcription factors such as bHLH, MYB, WRKY, NAC, ABI5, ARF, PIFs, and HY5 play crucial roles in regulating the *cis*-elements in *Prunus* species. These regulatory networks integrate environmental cues, hormone signaling, and developmental pathways, affecting carotenoid biosynthesis. In *Prunus* species, the expression levels of these genes can differ due to species-specific variations, leading to differences in carotenoid types and quantities. For example, higher expression levels of genes like *PSY* and *LCYB* have been correlated with increased carotenoid content in specific plum cultivars, demonstrating a direct link between gene function and carotenoid accumulation (Deng et al. 2023).

Several transcription factors and signaling pathways facilitate the transcriptional regulation of carotenoid biosynthesis genes, which can vary across species. Ethylene response factors (ERFs) can influence the expression of carotenogenic genes, thereby affecting the carotenoid profile. In *Prunus* species, different cultivars respond differently to ethylene, showing how regulatory mechanisms can form carotenoid accumulation based on each species'genetic makeup (Stanley and Yuan 2019). The variation in carotenoid accumulation among different *Prunus* species chiefly results from differences in gene function and the regulatory mechanisms affecting these genes. The relationship of biosynthetic pathways, gene expression patterns during fruit development, and regulatory networks contributes to the variation in carotenoid profiles among species.

The promoter regions of genes involved in carotenoid synthesis exhibit variations in their *cis*-regulatory elements. These *cis*-elements, which regulate gene expression by interacting with transcription factors, differ in type and arrangement across the promoter regions, potentially leading to variations in the expression patterns of the genes under specific conditions.

By examining these regulatory networks, researchers can gain deeper insights into the molecular processes responsible for the vibrant hues’ characteristic of *Prunus* fruits, facilitating targeted breeding strategies and crop improvement efforts. Consequently, the genus *Prunus* offers valuable genetic resources for analyzing new biochemical and molecular mechanisms driving carotenoid biosynthesis and accumulation and discovering novel genes. Understanding these complex interactions between environmental factors and genetic mechanisms is crucial for developing cultivation strategies that maximize carotenoid content in *Prunus* fruits.

### Future perspectives

Advanced molecular breeding and genome editing technologies, such as CRISPR-Cas9, offer extraordinary precision in manipulating carotenogenic genes, essential in carotenoid biosynthesis. Complementing these approaches, optimizing chromoplast development through genetic engineering could significantly enhance the sequestration, stability, and bioavailability of carotenoids in *Prunus* fruits. Chromoplasts, as specific plastids, play a critical role in carotenoid storage, and engineering their biogenesis and morphology could provide new opportunities for improving carotenoid accumulation. Additionally, it is crucial to explore the complex regulatory mechanisms governing carotenoid accumulation at multiple levels. Post-transcriptional and post-translational modifications, such as alternative splicing and protein–protein interactions, modulate enzyme activity and stability, adding control layers to the carotenoid biosynthetic pathway.

Comparative analyses across *Prunus* species, focusing on chromoplast development, gene expression, and plastid biology, could identify conserved and different patterns of carotenoid accumulation. These studies would provide an understanding of evolutionary adaptations, offering valuable information for breeding programs to improve fruit quality across multiple species. Investigating environmental factors, such as light intensity, temperature fluctuations, and nutrient availability, is equally crucial. Controlled experimental studies can illuminate how these external signals modulate carotenoid biosynthesis, guiding sustainable agricultural practices to maximize carotenoid content in *Prunus* fruits.

Moreover, the interaction between carotenoid biosynthesis and other metabolic pathways, including hormone signaling and stress response pathways, represents an exciting area for future research. For instance, hormones like gibberellins, abscisic acid, and cytokinin may influence carotenoid pathways directly or indirectly, creating chances to manipulate these interactions to enhance carotenoid accumulation. Stress responses like drought or heat may also impact carotenoid biosynthesis, providing paths for developing climate-resilient *Prunus* varieties. Lastly, integrating biotechnological advancements with synthetic biology offers a transformative approach to improving carotenoid traits. Designing regulatory networks to modify the expression of carotenogenic genes or enhance chromoplast biogenesis could significantly improve *Prunus* fruits'nutritional and aesthetic properties. Combining these strategies with computational modeling can optimize gene editing and metabolic engineering efforts.

## Data Availability

Not Applicable to this article as no datasets were generated or analysed during the current study.

## Abbreviations

GGPP: Geranyl–geranyl pyrophosphate synthase
PSY: Phytoene synthase
PDS: Phytoene desaturase
ZISO: ζ-Carotene isomerase
ZDS: ζ-Carotene desaturase
CrtISO: Carotenoid isomerase
LCYB: Lycopene α-cyclase
LCYE: Lycopene ε-cyclase
BCH: β-Carotene hydroxylases
CYP97: Cytochrome P450, Family 97
ZEP: Zeaxanthin Epoxidase
VDE: Violaxanthin de-epoxidase
NXS: Neoxanthin synthase
NCED: 9-Cis-epoxy carotenoid dioxygenase
MYB: The MYB transcription factor family, originally identified in the avian myeloblastosis virus
bHLH: Basic Helix-Loop-Helix transcription factor family
VOCs: Volatile organic compounds
DXS: 1-Deoxy-D-xylulose 5-phosphate synthase
TFs: Transcription factors
