# Correction: Carotenoid biosynthesis in *Prunus *species: from pathway and accumulation structure to diverse pigmentation

**DOI:** 10.1186/s43897-026-00242-x

**Published:** 2026-06-30

**Authors:** Naila Mir Baz, Jiahui Wang, Xulei Zhao, Asia Maqbool, Caizhen Gao, Pengfei Wang, Haijiang Chen, Hongbo Cao

**Affiliations:** College of Horticulture, Hebei Agricultural University, Baoding, 071000 China

**Correction: Mol Hortic 6, 3 (2026)**

**https://doi.org/10.1186/s43897-025-00188-6**

Following the publication of original article (Baz et al. 2026), it is reported that the Authors’ contributions, Funding, Data availability, Ethics approval and consent to participate, Consent for publication, Competing interest, and Acknowledgement sections do not correspond to the original manuscript due to inadvertent errors during production. The error does not affect the scientific content, findings, or conclusions of the article.

The original article (Baz et al. 2026) has been corrected.
